# Addressing female genital mutilation in Europe: a scoping review of approaches to participation, prevention, protection, and provision of services

**DOI:** 10.1186/s12939-017-0713-9

**Published:** 2018-02-08

**Authors:** Helen Baillot, Nina Murray, Elaine Connelly, Natasha Howard

**Affiliations:** 1 Formerly Scottish Refugee Council, 5 Cadogan Square, Glasgow, Scotland; 2 Department of Global Health and Development, Faculty of Public Health and Policy, London School of Hygiene and Tropical Medicine, 15-17 Tavistock Place, London, WC1H 9SH UK

**Keywords:** FGM, Female genital cutting, Gender based violence, Europe

## Abstract

**Background:**

Public and policy attention to female genital mutilation (FGM) in diaspora communities has increased in Europe, but research remains limited and misinformation abounds. As a first step to addressing these issues, this study explored FGM prevention and response interventions in Europe, using a scoping literature review and key informant interviews.

**Methods:**

A scoping study design was selected, using Arksey and O’Malley’s six-stage scoping framework to review identified sources. Key informant interviews were used to inform and add depth to literature findings. Findings were summarised thematically, guided by the Scottish Government’s ‘4Ps’ framework for tackling violence against women (i.e. participation, prevention, protection, providing services).

**Results:**

Seventy literature sources, of 1095 screened, plus 16 individual and 3 group interview sources were included. Several countries have developed promising interventions supporting FGM resistance and recovery. However, gaps remain including community participation, professional knowledge and linkages, and evaluation of approaches.

**Conclusions:**

This scoping review is an initial attempt to describe available primary evidence on European initiatives responding to FGM. Further research is required to determine whether interventions are effective, while policy and practice development must be shaped and driven by the experiences, needs, and views of affected communities.

## Background

Female genital mutilation (FGM) has affected millions of women and girls across continents, belief systems, and socioeconomic strata for approximately 5000 years. More than 125 million women and girls are affected today, predominantly across central Africa, parts of the Middle East and South Asia, and diaspora communities [44, 48]. While its origins are unclear, as populations become increasingly mobile, FGM occurs globally in diaspora communities [17].

FGM, defined by the World Health Organisation (WHO) as “*all procedures involving partial or total removal of the female external genitalia or other injury to the female genital organs for non-medical reasons*”, is internationally recognised as a violation of the fundamental rights of women and girls [50]. While different terms are used, including ‘cutting’ and ‘circumcision’, this article uses ‘FGM’ in recognition of the severity of harm caused.

*“The practice of FGM is an expression of deeply entrenched gender inequalities, grounded in a mix of cultural, religious and social factors inherent within patriarchal families and communities…The reported method, rationale and means of practising FGM are different in different communities, but FGM is fundamentally bound up with systems of patriarchy and… repression of female sexuality.”* [17].

Reported prevalence rates vary dramatically across and within countries. Highest reported prevalence rates are in Somalia (98%), Guinea (97%), Djibouti (93%), Sierra Leone (90%), and Mali (89%) [45]. In 50% of practising countries, girls undergo FGM before the age of five, while in the remainder, FGM is conducted on girls aged 5–14 [44]. UNICEF estimates 83 million survivors in Egypt, Ethiopia, Nigeria, and Sudan alone [44].

FGM can have multiple long and short-term physical and mental health consequences for those subjected to it and their communities [44]. Despite criticism of over-generalising the severity of its consequences [36, 42], research conducted amongst migrant women in Europe has generally confirmed that women living with FGM can experience recurrent sexual, psychological, and physiological problems [3, 46] and particularly before and during childbirth, women who have undergone the most severe forms are likely to require specialist surgical and psychological interventions [13].

This study aimed to explore FGM prevention and response interventions in Europe, using literature and interview sources. Study objectives were to: (i) describe EU interventions, using literature and in-depth key informant interview sources; (ii) assess whether interventions that appeared successful might be replicable, and (iii) propose promising interventions that could be implemented more widely.

## Methods

### Study design

A qualitative mixed-methods study design was selected, including a scoping literature review and key informant interviews to fill data gaps.

### Data collection

*Scoping review:* This was guided by Arksey and O’Malley’s six stage framework [4]. First, the research question was identified as “*Which FGM-related interventions have been tried in Europe that appear to have most potential to reduce or mitigate FGM among women and girls in affected communities?”* Research question selection was guided by the York framework, which recommends a broad, clearly articulated question, defining concept, target population, outcomes, and scope while accounting for the aim of the review [49]. Second, HB searched specialist databases on health, migration and gender systematically, i.e. PubMed, ScienceDirect, Intermid, EIGE, and IZA. Search terms, adapted for each database, included: ‘female genital mutilation’ [All Fields] AND/OR ‘female genital cutting’ [All Fields] AND/OR ‘female circumcision’ [All Fields] AND (“EU” [All Fields] OR “Europe” [MeSH Heading] OR “Europe*” [All Fields]) AND/OR ‘Norway’ AND/OR ‘Switzerland’ [All Fields]. Key journals that were hand-searched, were *Journal of Midwifery and Women’s Health; International Journal of Gynaecology and Obstetrics; Healthcare for Women International; Journal of Refugee Studies*; *European Journal of Migration and Law*; and *Culture, Health and Sexuality*. To ensure search comprehensiveness and further validate the approach, a four-stage search strategy was implemented: (i) publications posted on websites of well-known non-profit organizations working on FGM were searched; (ii) relevant citations were snowballed to references and websites of other pertinent organizations; (iii) a Google search of ‘female genital mutilation/cutting’ was conducted to include additional relevant documents; and (iv) stakeholder recommendations were assessed according to eligibility criteria [49]. Third, potential sources were screened against eligibility criteria (i.e. published or unpublished; primary or secondary research articles including more than one study participant; covering interventions in the EU, Norway, and/or Switzerland; available in a language the research team can read, i.e. English, French, Portuguese, or Spanish; published in 2007–2014). Fourth, data were charted in Excel using lead author name, source year, source type, study type, and the following deductive thematic headings and inductive sub-thematic headings: Protection (asylum, child protection, criminal justice); Prevention and Participation (awareness-raising & education, behaviour change, working with communities, role of professionals); Provision of services (obstetrics and gynaecology, reconstructive surgery, midwifery, psychological support, specialist clinics, other services). The four thematic headings correspond to the Scottish Government’s framework for tackling violence against women [41]. We identified this as an appropriate framework as we intentionally situated our analysis of FGM within the scope of violence against women. Additionally, this framework included ‘participation,’ while other relevant frameworks did not. Finally, as the research was conducted to inform Scottish government policy and the team had experience implementing this framework with migrant women, we concluded this was the best framework for us to choose. Fifth, the research team presented preliminary findings to 28 stakeholders and invited them to identify additional data sources that should be included. Sixth, data were summarised thematically.

#### Interviews

Key informants were identified for their expertise in EU-based FGM interventions in one or more ‘4P’ area and recruited purposively to provide a range of experiences, contexts, perceptions, and ideas for future work. The final sample included academics and professionals in Belgium, England, France, Ireland, the Netherlands, Scotland, and Spain working in health, policing, legal practice, NGOs, and government. Individual interviews were conducted in English or French, after written informed consent was provided. Five purposively-sampled group interviews were conducted; 3 with 28 senior and mid-level policy-makers, practitioners, and community representatives and 2 with representatives of community-based organisations from potentially-affected communities in Scotland, selected for their FGM expertise and activism. Individual and group interviews lasted approximately 1–1.5 h, were digitally-recorded or scribed, and professionally transcribed.

### Analysis

Transcripts were cleaned and coded using Dedoose software by NM and EC. Interview and literature sources were analysed thematically, with a realist perspective as described in Braun and Clarke, by NM and EC, with consistency checks by HB [9]. Data were categorised deductively by NM, EC and HB according to the four themes of the Scottish Government ‘4Ps’ framework, i.e. participation, prevention, protection, provision of services [41]. Within each broad deductive theme, sub-themes were identified inductively. For example, under Protection, we inductively identified sub-themes of asylum, child protection, and criminal justice. Additionally, an independent inductive theme of ‘data and research’ emerged. Discrepancies and divergent perspectives were discussed and agreed by all authors.

## Findings

### Sources

**Fig. 1:**
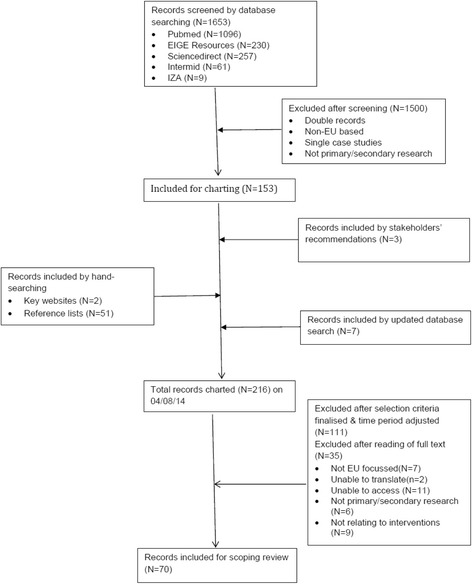
Scoping review flow diagram

Figure 1 provides a flow diagram of the literature search. Table 1 shows 70 literature sources included, 9 global, 8 EU-wide, and 53 covering one or more country. The UK was best represented (22 sources); then France (9); Sweden (6); Norway (4); Spain (3); Italy, Netherlands and Switzerland (2 each); and Belgium, Finland, and Germany (1 each). Table 2 shows interview sources, including 16 key informants participating in individual interviews (12 in-person, 4 Skype) and 35 participating in four group discussions.

**Table 1:** Scoping review sources, ordered by country and lead author

Lead author, year	Design	Participation	Prevention	Protection	Provision	Communities
Belgium (1)						
Dieleman, 2010 [14]	Focus groups/interviews^a^	x	x	x	x	Djibouti, Guinea, Somalia
Finland (1)						
Johansson, 2008 [20]	Project report	x	x			Somalia
France (9)						
Andro, 2010 [3]	Qualitative interviews				x	Guinea, Mali, Senegal,
Andro, 2009 [2]	Qualitative interviews		x		x	(as above)
Johnsdotter, 2009a [24] Johnsdotter, 2009b [25]	Cohort study				x	
Grunvald, 2008	Secondary research		x		x	
Le Roux, 2009	Qualitative interviews		x		x	
Martin, 2007 [32] Wehenkel, 2007 Njierboer, 2010	Secondary research		x	x		
Kool, 2014^b^	Secondary research			x		
Germany (1)						
Behrendt, 2011 [6]	Qualitative interviews^a^	x	x		x	Ghana, Nigeria, Togo
Italy (2)						
Caroppo, 2014	Survey			x	x	
Turrilazzi, 2007	Secondary research			x		
Netherlands (2)						
Vloeberghs, 2012 [47] Vloeberghs, 2011 [46]	Qualitative interviews^a^	x		x	x	Eritrea, Ethiopia, Sierra Leone, Somalia, Sudan,
	Qualitative interviews	x	x	x		(as above)
Norway (4)						
Gele, 2012a [19] Gele, 2012b [20] Lien & Schulz, 2014 [30]	Focus groups	x	x			Somalia
	Focus groups		x			Somalia
	Qualitative interviews		x	x		Somalia, Gambia, Eritrea, Kenya
Schulz, 2013	Qualitative interviews	x	x			Somalia, Gambia
Spain (3)						
Azkona, 2015	Proceedings paper		x			
Kaplan, 2009a	Survey of professionals			x	x	
Kaplan, 2009b	Project description	x	x			
Sweden (6)						
Isman, 2013	Qualitative interviews	x	x			Somalia
Foldes, 2012 [23]a	Qualitative interviews	x				Eritrea, Ethiopia
	Literature and case review			x		
Litorp, 2008	Survey/clinical review	x			x	Eritrea, Somalia
Lundberg, 2008	Qualitative interviews	x	x		x	Eritrea
Widmark, 2010	Qualitative interviews				x	
Switzerland (2)						
Abdulcadir, 2014 [1]	Review of clinical files				x	
Renteria, 2008	Literature review, clinical case studies		x		x	
UK (22)						
Bindel, 2014 [8]	Survey		x	x		
Brown, 2013 [10]	Participatory action research	x	x			
Creighton, 2010 [13]	Report		x		x	
Dustin, 2010 [10] Esmee Fairbairn Foundation, 2013 [16]	Secondary research	x		x		
Gordon, 2007	Clinical review/interviews				x	
	Secondary research			x		
Guine, 2007 [21] Hemmings, 2011 [22]	Report^a^	x	x			
Hussein, 2008	Observational data	x			x	Somalia
Jones, 2010	Qualitative interviews/ survey				x	
Lavender, 2009 [27]	Survey		x		x	
	Qualitative interviews	x	x		x	Somalia
Levac, 2010 [33]	Literature review & stakeholder interviews		x		x	
Liao, 2013 [29]	Qualitative participatory research^a^	x	x			Eritrea, Somalia, Sudan
Moore, 2014 [33] Norman, 2009 [35] Paliwal, 2014 RCM, 2013 [37] Relph, 2013 [39] Safari, 2013 [40] Strauss, 2009 Zaidi, 2007 Zenner, 2013	Retrospective case analysis			x	x	
	Questionnaire		x		x	
	Qualitative interviews				x	Somalia
	Qualitative interviews				x	Somalia
	Questionnaire				x	
	Retrospective clinical audit				x	
	Literature review/workshops		x	x	x	
	PEER, project report, qualitative interviews^a^	x	x			
	Conference report	x	x	x	x	
EU-wide (8)						
Berg, 2013 [7]	Literature review	x	X			
EIGE, 2013 [17]	Literature review	x	X	x	x	
Krasa, 2009 [31]	Literature review		x	x	x	
Leye, 2009a [34]	Survey/workshops				x	
Leye, 2009b	Questionnaires, workshops, expert reports			x		
Leye, 2009c	Literature review		X	x		
Leye, 2007	Questionnaire		X	x		
Novak, 2013	Statistical analysis		X	x		
Global (9)						
Balogun, 2012 [5]	Literature review				x	
Boyle, 2010	Secondary research^a^	x	X	x		
Costello, 2013 [11]	Literature review	x	X	x	x	
Cottingham, 2009 [12]	Secondary research		X	x		
Kontoyannis, 2010	Literature review		X		x	
Lien, 2013 [30]	Qualitative	x	x			
Mulongo, 2014	Literature review				x	
NMHES, 2009	Report	x	x	x		
Reig Alcaraz, 2014	Literature review	x	x	x	x	
	**totals**	***N*** **= 28**	***N*** **= 43**	***N*** **= 29**	***N*** **= 40**	***N*** **= 20**

NB: ^a^Included participatory research methods; ^b^Included the Netherlands

**Table 2:** Participant characteristics

ID	Role/Location	Participation	Prevention	Protection	Provision	Research
KIF01	Academia/Barcelona (Skype)		X	X		X
KIF02	NGO/Paris		X	X		
KIF03	NGO/Utrecht	X	X	X		X
KIF04	Education/Bristol	X	x			X
KIF05	Government/Amsterdam		X	X	X	
KIF06	Academia/Ghent			X	X	X
KIF07	Community/Dublin (Skype)	X	X			
KIF08	Medicine/London				X	X
KIF09	Academia/Nice				X	X
KIF10	INGO/EU	X	X	X		X
KIF11	Law/Glasgow			X		
KIM12	Police/London		X	X		
KIF13	Police/London		X	X		
KIF14	Law/Paris (unrecorded)		X	X	X	
KIF15	NGO/London	X	X		X	X
KIF16	Medicine/Glasgow				X	
EG1	28 participants/Glasgow	X	X	X	X	X
CG1	4 participants/Glasgow	X				X
CG2	4 participants/Glasgow	X				X

### Thematic analysis

Results are presented under deductive themes of participation, prevention, protection, and provision, and an inductive theme of data and research. Many results related to more than one theme. For example, protective interventions were often perceived to have a symbolic preventative effect (e.g. prosecuting perpetrators), by highlighting the illegality of FGM.

### Participation

Participation interventions aim to prevent FGM through awareness-raising or behaviour change within potentially-affected communities [30]. In total, 42% (28/66) of literature sources and all interview sources discussed participation. Two participation themes emerged (i.e. need for participation, lack of engagement). Community participation is further explored in another article (Connelly et al., in preparation).

#### Need for participation

Across all contexts, participants emphasised the importance of working *with* communities to ensure that policy-making and practice development was shaped by the experiences, needs, and views of those affected by FGM. A grassroots approach was seen as essential and even statutory service providers stressed “*Nothing will be achieved unless we secure the support of the community”* (KIF13).

#### Lack of engagement

Despite clear consensus on the importance of involvement by affected communities in any work to address FGM, participants noted a lack of ‘*work with communities’* on the part of governments (KIF01). The UK in particular was seen as lagging.

*“In Holland, you’ve seen the example of them being much more engaged with the communities and working with them on these issues. In the UK we tend to have a piecemeal approach.”* (KIF15).

This view was supported by the literature.

*“In the UK, efforts to reduce FGM have focused on punitive legislation without at the same time sufficiently empowering women in the communities concerned to engage in debate, change attitudes and create alternative ways of affirming their cultural identity.”* [15].

### Prevention

Preventive interventions aim to create and sustain behavioural and attitudinal change within affected communities. Two prevention themes emerged (i.e. awareness-raising, professionals’ role).

#### Awareness-raising

Participants stressed the need for concerted efforts to tackle FGM across multiple public-facing bodies encompassing the entire political spectrum, so awareness-raising could reach the general public, service providers, and policy-makers (KIF11). Multi-level approaches were considered beneficial, with UK participants reporting collaborative awareness-raising had pushed Government to respond to FGM (KIF01; KIF15). Several participants mentioned the Europe-wide *End FGM* campaign led by Amnesty International Ireland as an important approach at European level that enabled NGOs to be heard (EG1; KIF01; KIF02). Many stressed the importance of targeting FGM awareness-raising towards young people, particularly young women and girls (KIF13; KIF04; KIF15).

Participants described traditional media (e.g. television, radio) as having significant power in FGM work, confirming literature findings [2, 38, 46]. Impact was generally seen as beneficial, though working with media was sometimes noted as exhausting (KIF16).

#### Professionals’ role

Professionals can play a key role in longer-term FGM prevention, as affected women and girls contact services [11, 12, 26, 32, 33, 38]. Opportunities (largely missed) in health, education, social-work, and policing were highlighted.

Many participants discussed the preventive role of health professionals, particularly midwives, obstetricians, paediatricians, general practitioners (GPs), and school doctors. One spoke of work in Catalonia to train health and other professionals with links to potentially-affected families, suggesting this benefitted both families and professionals who felt “*empowered by knowledge*” to discuss FGM (KIF06). Participants identified GPs, as a universal contact point with a potentially key role in prevention (KIF08; KIF11). However, they were also identified as difficult to engage, mainly as FGM formed a minor part of their busy workload (KIF03; KIF16; KIF10; KIF05; KIF02), and minimal literature explicitly examines GP roles in FGM prevention. Maternity services were identified as a key awareness-raising intervention point with women from affected communities by participants and literature sources. However, it was unclear whether maternity professionals were ready to engage in prevention, or if “*most of the midwives don’t have any idea about the types of FGM or the consequences”* (KIF08). For example, a review of clinical notes, from a maternity unit attached to a specialist FGM clinic, found professionals failed to undertake preventative work with mothers who had undergone FGM and family members, such as explaining the illegality of FGM [51].

Education services were identified as having a key preventive role. An example in France incorporated FGM into the curriculum without stigmatising particular communities or children:

*“In Normandy, they’ve included [FGM] into a more general module around sexuality… reproductive health…they look at the particular issues of forced marriage and FGM. They explain that these issues concern large populations, without necessarily focussing on African countries, and show that these are quite general issues that can affect large numbers of people.”* (KIF03).

However, despite the significant potential role of teachers in FGM awareness raising and prevention, minimal research was found on teacher knowledge or attitudes towards FGM prevention work.

Very few participants explored a preventive role for social-workers, despite their potential contact with at-risk families (KIF06).

*“Social workers and health and welfare professionals have responsibilities...to protect girls from being cut; to advocate for services for affected women...and to engage with practising communities in processes to stop the practice.”* [11].

Policing could also go beyond criminal investigation to include partnerships in prevention, education, and awareness-raising, including other statutory professionals e.g. health and education (KIF13).

### Protection

Protection typically focuses on protecting the rights of individual women and girls. Interventions include child protection, risk assessment and reporting, the role of law, prosecutions, and international protection. Three protection themes emerged (i.e. gender-based violence, differing national approaches, prosecution).

#### Gender-based violence

Several participants noted the importance of contextualising FGM as a form of violence against women and girls, acknowledging the role of gender, power, and control in perpetuating the practice, and “*the need to situate FGM and analyse it within the wider continuum of honour-based violence”* (KIF13). For example, Dutch approaches incorporate FGM into existing work around child and domestic abuse, defining FGM as a “*special form of child abuse”* through a child rights lens (KIF03). Discussing FGM within the context of human rights, child abuse, and challenging patriarchy was considered important by some:

*“I was talking to a couple of women who did not know they had rights for anything. That’s why it’s important for them to understand the definition of human rights and also FGM is against human rights.”* (KIF02).

The importance of developing a Europe-wide understanding of FGM as a form of gender-based persecution was particularly emphasised to convey linkages to asylum adjudication and the Refugee Convention to decision-makers (KIS01). One described the development of UK asylum case law around FGM following the ‘*seminal*’ Fornah case (UKHL, 2006), which accepted that women from affected communities who had not undergone FGM could be considered at risk of persecution under the 1951 Refugee Convention (KIF11). This could explain the comparatively high recognition rate for asylum claims based on FGM in the UK compared to other European countries [43]. However, many EU countries were noted as not collating or publishing data on the grounds for asylum claims, making it impossible to monitor the number of FGM-based asylum claims. Belgium is an exception (KIS01; KIF06), and immigration officials play an active role in protection interventions. Any family granted leave to remain on the basis of a girl’s fear of FGM is invited annually to present to the asylum decision-making body a medical certificate confirming she remains ‘intact’ (KIF06; [14]). Sanctions are enforced if they do not respond. No evaluation of this initiative was found, so benefits versus potential ethical and administrative complexities remain unclear.

#### Differing approaches

A range of different national policies and practices aim to protect women and girls from FGM [17, 28]. This section synthesises participant views on different approaches.

#### Belgium

Many professionals “*don’t really have any sound knowledge on FGM*” (KIF02). However, the Flemish Forum for Child Abuse recently “*conducting a series of workshops…with the child protection sector…police and…educational sector to really see…what the gaps are*” (KIF02), led to submission of a briefing on FGM and child protection to responsible ministers and development of a new child protection protocol based on the Dutch model:

“There was a protocol the Netherlands developed. We invited key people from the Netherlands to hear, to discuss and to show us how they do it…” (KIF02).

Government funded specialist NGOs and academics to contribute to developing and delivering training for professionals and incorporated this into a national action plan to address FGM:

*“In the first phase we trained health professionals in one province that had proven in the prevalence study [to have] the highest concentration of women and girls from FGM risk countries...”* (KIF02).

#### France

With the most successful prosecutions for FGM in the EU, France is referenced as a ‘shining example’ of protection policies [8, 17, 21]. A French participant emphasised that the approach is not founded on principles of punishment but on the child’s best interests:

*“The child has to be at the centre […] governments are [not] interested in putting parents in prison. The best interests of the child take primacy over everything; it’s really about children’s rights, not women’s rights.”* (KIF02).

The French approach is two-pronged, combining preventive work with a strong legal and child protection framework. Increases in the number of families in France abandoning FGM was attributed to several high profile criminal trials (KIF10). The threat of prison was seen as a deterrent, and well-publicised trials at the highest judicial level were identified as having helped break down taboos (KIF10). Activists campaigned for the first FGM trials to be held at the *Cour d’Assizes* rather than tribunal level because (i) legally the injury was one of mutilation and should not be tried in an administrative court; and (ii) strategically it raised awareness of the act and its illegality, thus having a deterrent effect.

Integral to the French approach is routine physical examination - including genitalia - of girls up to age six, regardless of ethnicity, accessing specialist health clinics (*Centres de Protection Maternelle et Infantile*). While generally considered successful, some raised concerns it could (i) transfer risk to older girls; or (ii) if initiated in additional sites only for girls from specific communities, as some recommended, increase discrimination and racism [23].

*“Why is this measure being proposed to detect FGM and not sexual violence or sexual abuse of children, in all children? I mean the cases of sexual abuse of children [are] so huge compared to FGM…”* (KIF06).

French child protection laws have enshrined the concept of a ‘*shared secret’*, which requires professionals to share what would normally be confidential if it is in the child’s best interests (KIF03). However, professionals sometimes perceive this statutory duty as a *‘relative duty and balance it against professional secrecy provisions’* (KIF03), given the tremendous difficulty of assessing FGM risk. Training, guidance, and access to advice were considered critical to avoid legal and ethical confusion (Johnsdotter, 2010; [31]).

#### Netherlands

*Ketenaapak* (‘Chain Approach’) forms the core of a multi-disciplinary partnership approach to protecting girls from FGM, seen by some as more integrated than France’s approach, despite the lack of criminal prosecutions [34]. *Ketenaapak* was premised on a clear five-step guideline for professionals on when, how, and to whom to report concerns, and child protection referral to social services or the police as the final step. Reporting was centralised to 20 designated child and family abuse reporting centres, each with an FGM expert to conduct risk assessments and support safeguarding measures. Key to the success of this unique approach was professionals feeling empowered to report concerns, involvement of affected communities, and a focus on supporting parents to protect their daughters, rather than as potential perpetrators (KIF05). Reporting FGM cases is not mandatory in the Netherlands, although professionals have a statutory *right to report* (KIF05)*.*

#### Spain

KIF01 described a ‘*repressive*’ police approach to safeguarding girls from FGM in Catalonia:

*“…they go door-by-door knocking at the doors and taking the girls’ passports off the parents and they have to have genital checking every six months until they’re 18 years […] As soon as they can send the girl, they will send her… This is not the way to do prevention, prevention is not for police.”* (KIF01).

The role of law enforcement officers in child protection was described as important but the *‘semi-final level,’* while a protocol-based preventive approach that trained professionals already in contact with families seemed more appropriate. Without community engagement and trust-building between families and professionals, reliance on the threat of imprisonment was deemed “*very dangerous”* (KIF01).

*“You don’t need to invent another circuit, they’re normally in contact because of their work with the families so if you train them, they will transfer this knowledge... We believe this is an evidence-based approach, a respectful approach…”* (KIF01).

#### United Kingdom

Participants indicated more progress could be made in developing a consistent and effective protection approach. A major challenge identified was lack of training and guidance, particularly around reporting [27, 28].

*“[*There is a*] disconnect between safeguarding children who are safeguarded from other forms of harm and safeguarding children from FGM*… *Over and over again you get a situation where safeguarding professionals… go to training [and] there’s no mention of FGM…. So professionals do not see it as part of their role or responsibility…”* (KIF15).

For example, recent Intercollegiate Guidance on FGM [37] was identified as unhelpful in the context of reporting adult survivors:

*“On one page it says all women should be referred to the police and the next page it says a referral should be considered with the consent of the women. So it has two completely different policies within the same document…”* (KIF16).

Suggestions included (i) making better use of existing structures and frameworks (e.g. Multi-Agency Safeguarding Hubs) to manage sensitive information appropriately and improve decision-making; and (ii) developing training, guidance, and resources for professionals to coordinate protection responses. For example, police in England referenced work to develop pathways, training and support for officers, investigators, prosecutors, and other professionals (KIM12).

*“I don’t think it’s because people don’t want to do it, they don’t know how to do it or they don’t know they should be doing it… [We need a] really clear referral service for when there’s suspicion that a child’s had FGM.”* (KIF16)

#### Prosecution

Participants highlighted barriers to prosecutions, some of which could be addressed through a victim-centred approach, training, awareness-raising among professionals and affected communities, a focus on combining a strong legal framework with investment in prevention, and development of guidance and frameworks for reporting and information sharing. Participants agreed that to protect women and girls from FGM, laws prohibiting and criminalising the practice must be enacted.

The law can serve to set out a protective framework that can be relied upon by affected communities, professionals, police, and the courts. However, opinions differed on its role in protection. Participants and sources emphasised that enacting legislation criminalising FGM was insufficient, as communities must be aware of the law for it to be effective (Johnsdotter, 2010; Boyle, 2010; [11]) and professional responses should be coordinated.

*“There are certain organisations that see the groups at risk. [*Asylum advice services are*] one, primary schools are another, hospitals, GP surgeries [...*It*] needs the courage to speak out and speak openly about this and cut through the cultural taboos.” (*KIF11).

Participants highlighted the need for a victim-centred ‘*violence against women and girls*’ approach to prosecutions, to strike the correct balance between needs of victims and the need to eradicate FGM (KIF13).

Grassroots projects critiqued the lack of UK prosecutions, suggesting a protection gap still existed (Integration Bristol, 2012). One reason for the lack of UK prosecutions was noted as because the police engaged in proactive intervention and prevention work, speaking to affected communities and professionals (KIF12). A key prosecution barrier was the likelihood a victim would need to testify against her relatives and balancing this against the child’s best interests ([8]; KIF15; KIM12; KIF13; KIF01).

*“I think there’s a need for the police service to provide confidence to other professionals around how we’re going to handle sensitively and confidentially that information.”* (KIF13).

Another potential barrier was a lack of understanding about FGM and affected communities among police officers (KIF03). NGOs were noted as playing an important role breaking down some of these barriers though training and awareness-raising, thus countering reliance on “*thin ethnic and cultural evidence*” that bolstered perceived discrimination in some communities [31].

*“We’ve been very instrumental in training the police to have a better understanding of FGM and be able to really understand the challenges around FGM as a special form of child abuse.”* (KIF15).

### Providing services

Service provision consists largely of specialist clinical and psychosocial responses for FGM survivors. Three themes emerged (i.e. access, ‘reconstructive’ surgery, and sustainability).

#### Access

Health services were considered the main entry point to support for FGM survivors, who often only engaged with services when pregnant. However, while maternity services were potentially important in facilitating access, overreliance risked disregarding the needs of survivors who were not pregnant or of childbearing age.

A significant barrier was survivor reluctance to approach services for fear of a negative reaction from health professionals (KIF05) or their own feelings of discomfort [1]. For example, some communities in the Netherlands reportedly believed mistakenly that being a survivor of FGM was illegal and so were afraid to disclose (KIF05). However, in a specialist London service, although self-referrals remained low at 5–10%, increasing numbers of younger women self-referred for de-infibulation advice “*because they’re taking charge of their lives”* (KIF16).

Barriers to response and referral arose from cultural sensitivity and lack of knowledge among professionals. The literature suggests increased cultural competence could improve provision of appropriate services for women from affected communities.

*“Culturally competent care must surpass tolerance and good deeds, moving towards greater respect and acceptance of the similarities and differences between cultures, where increased understanding can improve communication and facilitate positive clinical outcomes.”* [33].

Multi-disciplinary health responses to FGM were seen as key by many participants. For example, a specialist London centre offered ‘*three or four clinics a month… with outpatient de-infibulation procedures…inpatient surgeries…assessment by a midwife for safety for delivery… a team approach’* (KIF16). In Belgium, a specialist clinic offers women ‘*10 consultations, not only for clinical aspects but also for psychological and sexual aspects*’ (KIF01). In France, ‘*a woman will be assessed by a multi-disciplinary team including a gynaecologist, a sexologist, a psychologist before being offered [a surgical] procedure’* (KIF09). In the Netherlands, ‘*[in] all six places [*offering specialist services*] there is a team behind it: sexologist, psychologists, gynaecologists, midwives*’ (KIF05). Participants particularly agreed on the need for psychological and psychosexual services for survivors. However, psychological support was identified as a significantly under-resourced and under-researched area of survivors’ service provision [3, 13, 29, 40].

#### Reconstruction

Participants were more divided on provision of ‘reconstructive’ surgery for FGM survivors. French participants indicated that availability of this surgery was useful to ‘*break the silence*’ around FGM within families and between clinicians and patients [3]. However, UK participants particularly reported concerns about the lack of evidence on available surgical procedures. Community representatives were positive about surgical developments, suggesting surgery should be offered to survivors where available. However, a health expert felt that until better studies than Foldes et al. are available [18], such procedures should only be offered as ‘cosmetic’ procedures:

*Cosmetic surgery is of benefit to women of course but then you should be clear and say this is a cosmetic procedure that will make it look better and you might have some benefit… until there are better studies around to show [reparative surgery] works and how it works and that it’s safe for women, it’s really not appropriate to be recommending it on a widespread basis.* (KIF16).

#### Sustainability

This was an issue for all interventions. Community-based organisations, though regularly approached for their expertise, were rarely funded for this advisory role and/or overlooked for government funding in favour of larger national charities (KIF15). A general need for longer-term investment, beyond development of protocols, frameworks, and action plans (KIF06; KIF10; KIF15), meant initiatives tended to rely on interested and committed individuals at both service and policy level.

*The difficulty with all new services is they’re often very dependent on the clinician that’s leading them...If you have a really…enthusiastic committed midwife, obstetrician or gynaecologist, then the services will develop… A keen midwife will run the service for two years...then she’ll leave...and it all fizzles out.* (KIF16).

### Data and research

Key areas identified by participants and literature sources as requiring further data and research were data collection, participatory methods, intervention outcomes, and professional roles in prevention.

#### Data

Participants discussed lack of either routine statistical or qualitative research data, particularly in health, asylum, and criminal justice. While pockets of good practice were identified, participants indicated that merely collecting data was insufficient as analysis and dissemination were needed to ensure appropriate monitoring of FGM and responses (KIF15). A number of issues with statistical data collection in health services in the UK were highlighted, ranging from problems identifying FGM, to coding and recording different types of FGM.

*“In England there are no standard obstetric notes [...] The questions are often not compulsory so if it’s not ticked… you don’t know if… it’s just not been asked.”* (KIF16).

However, even where codes for FGM existed, accuracy depended on professionals recognising and recording it correctly (KIF16). Even doctors working in ethnically diverse areas demonstrated a lack of confidence in identifying FGM [38, 51]. In Belgium, hospital recording improved following introduction of coding and descriptive information cards for different types of FGM (KIF06).

#### Participatory research

Participants described evidence of developments in peer and participatory research methods as tools for informing and developing interventions (KIF15). For example, Replace interventions (www.replacefgm2.eu/) were identified as innovative and positive contributions to peer research grounded in a theoretical behaviour change framework. Participatory methods enabled practitioners to fully involve affected communities [16] and highlight some complexities of FGM work, e.g. potential negative reactions peer interviewers faced within their communities:

*“The fact that some interviewers experienced their social network weaken can be considered one of the most substantial difficulties faced by the project.”* [6].

However, limitations still need to be addressed. First, with few notable exceptions [6], sample sizes tended to be self-selecting and unrepresentative, drawn from community groups already addressing FGM [46]. Second, research has focused on certain diaspora communities, particularly Somali and Eritrean. For example, of 21 studies included, 15 referenced or interviewed Somali women while 8 referenced the Eritrean community. None referenced Egyptian women, despite a national FGM prevalence in Egypt of 87% [45] and an estimated European diaspora of up to 790,000 (Migration Policy Centre, 2013). This likely reflects researcher access, interests and community links. However, it may also indicate a tendency to typify FGM as a concern among sub-Saharan African women, likely to arrive in Europe as asylum-seekers, rather than focusing equally on groups who may have entered Europe as students or employees.

#### Intervention outcomes

Participants noted the lack of follow-up research on health interventions [5]. Reviews have detailed numbers of service-users, but lack data on service-user satisfaction or outcomes. No clinical trial data were found, which may have been due primarily to the ethical challenges of researching surgical procedures in this context or to the perceived lack of funding for FGM research noted by participants. Surgical procedures were particularly under-researched.

*“Things like de-infibulation before delivery or in labour...there’s no guidance as to what’s best, so we do what we think is clinically best and sensible but there’s no evidence base at all for any of this.”* (KIF16).

#### Professional roles

Minimal research was identified on the preventative and protective roles of non-healthcare professionals. Only four studies focused on the potential preventative role of social care assistants (Caroppo, 2014), social workers [11], teachers (Azkona, 2015), and asylum officials (Novak, 2013). The role of education professionals was particularly under-researched, with no publications found on teachers’ knowledge, perceptions, or impact. This contrasted with participants frequently highlighting that teachers at all levels should have an important role across all domains of identification, protection, prevention and behaviour change within communities.

## Discussion

### Primary findings

Despite interest, minimal good-quality evaluation research exists on FGM interventions in Europe. This scoping study found some promising initiatives to work with diaspora communities in addressing FGM. Some countries (e.g. France) have led with strong criminal justice responses, while others (e.g. the Netherlands) have focused on preventative child protection measures. National approaches will likely continue to differ, because of differing criminal justice systems and concepts of citizenship and community [72].

### Implications for policy and practice

Despite a need for further evaluation, common themes that appeared to underlie successful approaches include recognition of FGM as gender-based violence, providing clear preventive roles for frontline professionals, clear protection and prosecution approaches, and participation of affected communities. Governments, statutory bodies, non-state actors (e.g. NGOs), and communities all have a role in advocacy and action.

At the policy level, we urge the EU and all national governments to recognise FGM as a form of gender-based violence, linked to other forms of violence against women and girls (e.g. forced marriage, honour killings). An approach that firmly situates FGM as a violation of women’s and girl’s rights can enable both professionals and concerned individuals within communities to overcome potential arguments about FGM being a ‘cultural practice’ that should not be discussed or addressed. For example, Dustin provides a discussion on interpreting such a ‘cultural imperative’ [15]. This is vital both to prevent FGM and to enable survivors to speak out more freely.

We urge governments to provide national direction by integrating FGM considerations into existing frameworks that tackle violence against women and girls. Governments can develop, and resource, national strategy and action-plans on FGM, including investments in supporting affected communities to create long-term behaviour change. Such action-plans should provide clear guidance on consistent recording of FGM indicators by statutory services, and promote interventions that are evidence-based and robustly evaluated. Given the current lack of robust evaluation, plans should also provide support for robust research.

In practice, all professionals have a potential role in FGM prevention that should be supported. We recommend that governments provide clear, national-level direction on the role of frontline professionals in FGM prevention and mitigation enabling relevant professional bodies and agencies to develop training on FGM for frontline staff. Work in schools can be particularly successful, but lessons should be drawn from projects that have successfully integrated preventative work into wider topics of equity and gender rights, to avoid stigmatising communities or individual children (for example https://federationgams.org/). Integrated, specialist services that include psychological support and offer a single entry-point, can be successful if well-resourced and not solely reliant upon certain individuals (KIF05, KIF09, [13]).

Protection by criminal justice and child protection must be enacted effectively and fairly. Social welfare and police forces must ensure that the criminal justice response is perceived as effective and that anyone found to have subjected a child to FGM will face robust criminal sanctions. Professionals from all sectors must have clear and accessible risk assessment and reporting guidelines.

Service provision should be assessed and designed using a cultural competency lens, to ensure services are accessible and useful to women and girls affected by FGM. Health services should consider establishing a specialist, multi-disciplinary FGM service in key areas, with clear links to named professionals across their territory. Relevant professional bodies should ensure that health professionals are trained to carry out sensitive inquiry about FGM and that all pregnant women are asked about FGM during standard obstetric and maternal health checks.

Participation with communities is vital to all interventions. Without a genuine and effective commitment to community participation, decision-makers will not understand the true levels of risk experienced by women and girls and risk further marginalising those community voices that are the most effective advocates for change. Interventions addressing FGM at community level require long-term sustainable investment.

### Limitations

First, a scoping review only includes studies within authors’ search and language capacities. Second, authors did not assess evidence quality, as scoping reviews include the broadest range of evidence from published and unpublished literature. Thus, findings should be interpreted accordingly. Third, due to funding and time restrictions, participant numbers and locations in this study were limited. Finally, focus on European interventions ignored the wealth of interventions successfully designed and implemented by African women within their own communities, including in Senegal (e.g. TOSTAN project www.tostan.org), Gambia, and Sudan, which offer international benchmarks for changing attitudes and limiting FGM.

## Conclusions

FGM is emotive and questions about the extent to which girls in diaspora communities in Europe are at risk of FGM have attracted substantial media interest. However, without improvements in data collection across statutory services and further research, particularly with potentially-affected communities, it remains difficult to quantify the extent of potential issues in individual countries. Despite data gaps, several European countries have begun developing interventions to support women and girls living within their borders to resist and recover from FGM.

## References

[1] Abdulcadir J, Dugerdil A, Boulvain M, Yaron M, Margairaz C, Irion O, Petignat P (2014). Missed opportunities for diagnosis of female genital mutilation. Int J Gynecol Obstet.

[2] Andro, A, Lesclingand, M and Pourette, D (2009) Volet qualitatif du projet Excision et Handicap (ExH): Comment orienter la prévention de l’excision chez les filles et jeunes filles d’origine Africaine vivant en France: Une étude des déterminants sociaux et familiaux du phénomène, Universite Paris and INED.

[3] Andro A, Lesclingand M, Pourette D (2010). Excixion et cheminement vers la reparation: une prise en charge chirurgicale entre experience personnelle et dynamiques familiales. Sociétés contemporaines.

[4] Arksey H, O’Malley L (2005). Scoping studies: towards a methodological framework. Int J Soc Res Methodol.

[5] Balogun OO, Hirayama F, Wariki WMV, Koyanagi A, Mori R. Interventions for improving outcomes for pregnant women who have experienced genital cutting. Cochrane Database Syst Rev. 2013;(2). Art. No.: CD009872. doi:10.1002/14651858.CD009872.pub2. 10.1002/14651858.CD009872.pub2 PMC7388007 23450610

[6] Behrendt, A (2011) *Listening to African Voices. Female Genital Mutilation/Cutting among Immigrants in Hamburg: Knowledge, Attitudes and Practice*, Plan International, Germany.

[7] Berg RC, Denison E (2013). A tradition in transition: factors perpetuating and hindering the continuance of female genital mutilation/cutting (FGM/C) summarized in a systematic review. Health Care Women Int..

[8] Bindel, J (2014) *An Unpunished Crime: The lack of prosecutions for female genital mutilation in the UK*, The New Culture Forum, London.

[9] Braun V, Clarke V (2006). Using thematic analysis in psychology. Qual Res Psychol.

[10] Brown, K, Beecham, D and Barrett, H (2013) The Applicability of Behaviour Change in Intervention Programmes Targeted at Ending Female Genital Mutilation in the EU: Integrating Social Cognitive and Community Level Approaches, Obstetrics and Gynaecology International 2013. 10.1155/2013/324362 PMC3745976 23983698

[11] Costello S, Quinn M, Tatchell A, Jordan L, Neophytou K (2015). In the best interests of the child: preventing female genital cutting (FGC). Br J Soc Work.

[12] Cottingham J, Kismodi E (2009). Protecting girls and women from harmful practices affecting their health: are we making progress?. Int. J. Gynaecol. Obstet..

[13] Creighton S (2010). Report from the harmful traditional practices and human trafficking sub-group: responding to violence against women and children – the role of the NHS.

[14] Dieleman M (2010). *Excision et Migration en Belgique francophone: Rapport de recherche de l’Observatoire du sida et des sexualités pour le GAMS Belgique*, Ed.

[15] Dustin M (2010). Female genital mutilation/cutting in the UK: challenging the inconsistencies. Eur J Women's Stud.

[16] Esmée Fairbairn Foundation (2013) *Tackling Female Genital Mutilation in the UK: What works in community-based prevention work*, London.

[17] European Institute for Gender Equality (EIGE) (2013). Female genital mutilation in the European Union and Croatia.

[18] Foldes P, Cuzin B, Andro A (2012). Reconstructive surgery after female genital mutilation: a prospective cohort study. Lancet.

[19] Gele AA, Kumar B, Harslof Hjelde K, Sundby J (2012). Attitudes toward female circumcision among Somali immigrants in Oslo: a qualitative study. Int J Women’s Health.

[20] Gele AA, Johansen EB, Sundby J (2012). When female circumcision comes to the west: attitudes toward the practice among Somali immigrants in Oslo. BMC Public Health.

[21] Guiné A, Moreno Fuentes FJ. Engendering Redistribution, Recognition, and Representation: The Case of Female Genital Mutilation (FGM) in the United Kingdom and France. Politics & Society. 2007;35:477.

[22] Hemmings, J (2011) *The FGM Initiative: interim report*, Options UK.

[23] Johnsdotter, S (2009) *The FGM Legislation Implemented: Experiences from Sweden*, Malmo University.

[24] Johnsdotter S, Moussa K, Carlbom A, Aregai R, Essén B (2008). “Never my daughters”: a qualitative study regarding attitude change toward female genital cutting among Ethiopian and Eritrean families in Sweden. Health Care Women Intl.

[25] Kaplan Marcusán A, López Gay A. Mapa de la Mutilación Genital Femenina en España 2009. Barcelona: Universitat Autònoma de Barcelona; 2009.

[26] Krasa K (2010). Human rights for women: the ethical and legal discussion about female genital mutilation in Germany in comparison with other western European countries. Med Health Care Philos.

[27] Lavender R (2009). Female genital mutilation in a globalized age. Br J Midwifery.

[28] Leye, E and Sabbe, A (2009) *Responding to Female genital mutilation: striking the right balance in Europe between prosecution and prevention. A review of legislation*, International Centre for Reproductive Health, Ghent, Belgium.

[29] Liao LM, Elliott F, Ahmed F, Creighton SM (2013). Adult recall of childhood female genital cutting and perceptions of its effects: a pilot study for service improvement and research feasibility. J Obstet Gynaecol.

[30] Lien, IL and Shultz, JH (2013) *Internalizing Knowledge and Changing Attitudes to Female Genital Cutting/Mutilation*, Obstetrics and Gynecology International Volume 2013. 10.1155/2013/467028 PMC3694526 23843795

[31] Lien IL, Shultz JH (2014). Interpreting signs of female genital mutilation within a risky legal framework. Int J Law Policy Family.

[32] Martin C (2007). Compte-Rendu de Colloque/Report on Scientific Meeting: Les mutilations sexuelles féminines. Theol Sex.

[33] Moore K (2014). *Female Genital Mutilation and Cultural Competency: Moving towards improved management of obstetric care*, unpublished Msc thesis.

[34] Nijboer, JF, Van der Aa, NMD and Buruma, TMD (2010) *Criminal Investigations and Prosecution of Female Genital Mutilation: The French Practice: Crime, Law Enforcement and Safety,* Ministry of Justice, Netherlands.

[35] Norman, K, Hemmings, J, Hussein, E and Otoo-Oyortey, N (2009) *FGM is always with us. Experiences, Perceptions and Beliefs of Women Affected by Female Genital Mutilation in London: Results from a PEER Study*, Options Consultancy Services and FORWARD.

[36] Obermeyer CM (1999). Female genital surgeries: the known, the unknown, and the unknowable. Med Anthropol Q.

[37] RCM, RCN, RCOG, Equality Now, UNITE (2013). Tackling FGM in the UK: intercollegiate recommendations for identifying, recording, and reporting.

[38] Reig Alcaraz, M, Siles Gonzalez, J and Solano Ruiz, C (2013) *Attitudes towards female genital mutilation: an integrative review*, International Nursing Review. 10.1111/inr.12070 24237176

[39] Relph S, Inamdar R, Singh H, Yoong W (2013). Female genital mutilation/cutting: knowledge, attitude and training of health professionals in inner city London. Eur. J. Obstet. Gynecol. Reprod. Biol..

[40] Safari F (2013). A qualitative study of women’s lived experience after deinfibulation in the UK. Midwifery.

[41] Scottish Government (2009) *Safer Lives: Changed Lives. A Shared Approach to Tackling Violence Against Women in Scotland*, Edinburgh.

[42] Shell-Duncan B (2008). From health to human rights: female genital cutting and the politics of intervention. Am Anthropol.

[43] UNHCR (2013) *Too Much Pain. Female Genital Mutilation and Asylum in the European Union: A Statistical Overview*, UNHCR.

[44] UNICEF (2013). Female genital mutilation/cutting: a statistical overview and exploration of the dynamics of change.

[45] UNICEF (2016) Percentage of women and girls aged 15–49 who have undergone FGM/C, http://data.unicef.org/child-protection/fgmc.html (accessed 16 Oct 2017).

[46] Vloeberghs, E, Knipsheer, J, van der Kwaak, A, Naleie, Z, and van den Muijsenbergh, M (2011) Veiled pain: a study in the Netherlands on the psychological, social and relational consequences of female genital mutilation, Foundation Pharos, Utrecht, Netherlands.

[47] Vloeberghs E, van der Kwaak A, Knipscheer J, van den Muijsenbergh M (2012). Coping and chronic psychosocial consequences of female genital mutilation in the Netherlands. Ethnicity Health.

[48] Whitehorn, J, Ayonrinde, O & Maingay, S (2002) *Female genital mutilation: cultural and psychological implications*, Sexual and Relationship Therapy, Vol.17:2.

[49] Woodward A, Howard N, Wolffers I (2014). Health and access to care for undocumented migrants living in the European Union: a scoping review. Health Policy Plan.

[50] World Health Organisation (WHO) (2014), *Female genital mutilation: Fact sheet N°241*, February 2014 http://www.who.int/mediacentre/factsheets/fs241/en (accessed 16 Oct 2017).

[51] Zenner N, Liao LM, Richens Y, Creighton SM (2013). Quality of obstetric and midwifery care for pregnant women who have undergone female genital mutilation. J Obstet Gynaecol.

